# NIR Spectroscopy for Predicting Physicochemical and Functional Quality Attributes of Berry (Aronia, Haskap, and Goji) Fruits

**DOI:** 10.3390/foods15152679

**Published:** 2026-07-29

**Authors:** Juan Carlos Solomando, Patricia Calvo, María José Rodríguez, Noelia Nicolás, María Ramos, Lucía León, Alberto Ortiz

**Affiliations:** 1 Centro de Investigaciones Científicas y Tecnológicas de Extremadura (CICYTEX), Instituto Tecnológico Agroalimentario de Extremadura (INTAEX), Área de Postcosecha, Valorización Vegetal y Nuevas Tecnologías, Avenida Adolfo Suárez s/n, 06007 Badajoz, Spain; jcsolomandog@gmail.com (J.C.S.); mariajose.rodriguezg@juntaex.es (M.J.R.); 2 Centro de Investigaciones Científicas y Tecnológicas de Extremadura (CICYTEX), Centro de Agricultura Ecológica y de Montaña (CAEM), Área de Fruticultura Mediterránea, Avenida España 43, 10600 Plasencia, Spain; noelia.nicolas@juntaex.es (N.N.); maria.ramos@juntaex.es (M.R.); 3 Centro de Investigaciones Científicas y Tecnológicas de Extremadura (CICYTEX), Instituto de Investigaciones Agrarias “Finca La Orden”, Área de Calidad de la Carne y Productos Cárnicos, A5 km 372, 06187 Badajoz, Spain; lucia.leon@juntaex.es (L.L.); alberto.ortiz@juntaex.es (A.O.)

**Keywords:** non-destructive analysis, portable spectrophotometer, emerging red berries, aronia, haskap, goji

## Abstract

This study evaluated the potential of a miniaturized portable near-infrared spectroscopy (NIRS) device for the non-destructive prediction of the physicochemical and functional quality attributes of red berries. A total of 145 samples from three berry species (aronia, haskap and goji), representing different harvest years and ripening stages, were analyzed. Spectra were acquired over the 908–1676 nm range using a MicroNIR™ 1700 OnSite-W spectrophotometer, and partial least squares regression models were developed to predict total soluble solids, moisture content, pH, total phenolic content and antioxidant capacity. The calibration models achieved coefficients of determination in cross-validation (R^2^CV) ranging from 0.83 to 0.92, with Root Mean Square Error of Cross-Validation (RMSECV) between 0.281 for pH and 2.085 g Trolox kg^−1^ FW for antioxidant capacity. External validation confirmed the robustness of the models, yielding R^2^EV values between 0.76 and 0.88 and Root Mean Square Error of Validation (RMSEV) ranging from 0.381 for pH to 2.095% for moisture content. The highest predictive performance was obtained for total soluble solids (R^2^EV = 0.88; RMSEV = 1.391), followed by moisture content, pH and antioxidant capacity, whereas total phenolic content showed the lowest predictive accuracy (R^2^EV = 0.76; RMSEV = 1.914 mg GAE g^−1^ FW). The Residual Prediction Deviation (RPD) and Range Error Ratio (RER) values further supported the practical applicability of the models for approximate quantitative prediction. Overall, these results demonstrate that portable NIRS is a rapid, non-destructive and reliable tool for the integrated assessment of the physicochemical and functional quality attributes of emerging red berry species.

## 1. Introduction

The cultivation of red berries has expanded considerably in recent decades, becoming one of the most dynamic and economically important sectors of modern horticulture. This group of fruits includes a wide range of species, such as blueberries, raspberries, mulberries, grapes, strawberries, and cranberries [1]. Their high commercial value, increasing global demand, and well-established nutritional and functional properties have attracted substantial scientific and industrial interest [2]. Berries are particularly rich in bioactive compounds, including anthocyanins, polyphenols, flavonoids, vitamins, and minerals, which have been associated with numerous health benefits such as antioxidant activity, improved metabolic regulation, enhanced immune function, and a reduced risk of chronic diseases [3]. Consequently, consumer demand and consumption of these fruits have increased significantly in recent years, reflecting growing awareness of their nutritional and functional value [4].

In addition to these widely cultivated species, less common berries such as aronia, haskap, and goji are attracting increasing attention as emerging and complementary crops in modern horticulture. These species are valued not only for their high content of health-promoting compounds [5] but also for their potential to diversify agricultural production systems. Such diversification is particularly relevant in the current context of climate change, where improving the resilience and adaptability of cropping systems has become essential for sustainable agricultural production [6]. Furthermore, many of these species exhibit remarkable tolerance to adverse environmental conditions, making them well suited for cultivation in marginal lands and mountainous regions, where conventional crop production is often constrained [7].

From an economic perspective, berry production represents a valuable opportunity for many agricultural regions. Compared with many traditional crops, berries generate high returns per unit area, making them particularly attractive to producers with limited land resources. In addition, the berry sector creates substantial employment opportunities throughout the value chain, including planting, harvesting, sorting, packaging, and distribution, all of which require intensive labor [8]. Consequently, berry cultivation contributes to rural livelihoods and stimulates regional economic development. In countries with favorable agroclimatic conditions, the production and exports of fresh and processed berries have also become important drivers of income generation and international market competitiveness.

Technological innovation has played a key role in the expansion of the berry industry, with near-infrared spectroscopy (NIRS) emerging as one of the most valuable tools for quality assessment. Combined with advanced chemometric methods, NIRS has become one of the most promising analytical techniques in the food industry, offering rapid, non-destructive, and reagent-free analysis of agricultural products [9]. Its application to quality control enables the rapid evaluation of multiple physicochemical and functional attributes, contributing to the production of consistent, safe, and high-quality foods [10]. In particular, NIRS allows the simultaneous prediction of parameters such as total soluble solids, moisture content, firmness, acidity, dry matter, and bioactive compounds including anthocyanins and total phenolics [11,12]. These advantages are especially relevant for berries, which are highly perishable fruits characterized by a short postharvest life and considerable variability in ripening stage and quality attributes [13]. The technique also enables real-time monitoring during harvesting, postharvest handling, storage, and processing, facilitating the classification of fruits according to maturity, internal quality, and intended market without damaging the product. Furthermore, NIRS can enhance traceability systems and support precision agriculture by helping producers optimize harvest timing and crop management decisions [14,15]. Compared with conventional laboratory methods, which are generally destructive, labor-intensive, time-consuming, and reagent-dependent, NIRS substantially reduces analysis time, operating cost and chemical waste generation while maintaining high analytical efficiency [9].

Previous studies on berries have primarily focused on hyperspectral imaging for the non-destructive prediction of internal quality attributes. Overall, the highest predictive performance has consistently been achieved for soluble solids content, whereas the prediction of functional compounds, such as total phenolics, anthocyanins, and antioxidant capacity, has generally been less accurate and more variable. For example, Fatchurrahman et al. [16] reported high predictive performance for soluble solids content and titratable acidity in fresh goji berries using hyperspectral imaging. These authors reported external coefficients of determination of up to 0.94 for soluble solids content and 0.84 for titratable acidity in fresh goji berries, while other parameters related to bioactive compounds showed lower or more limited prediction accuracy. Similarly, Seki et al. [17] obtained coefficients of determination above 0.85 for soluble solids prediction in strawberries using Vis–NIR and NIR spectroscopy [17], while Qiu et al. [18] reported prediction coefficients above 0.86 for soluble solids content in blueberries using NIR hyperspectral imaging. Despite these promising results, previous studies have largely focused on single species and a limited number of quality traits, particularly those related to sugar content. The novelty of the present study lies in the development of global multi-parameter PLSR models using a miniaturized portable NIR spectrophotometer for the simultaneous prediction of multiple physicochemical and functional quality attributes of three emerging berry species: aronia, haskap, and goji. Unlike previous studies, the calibration dataset combined samples from different species, harvest years, and ripening stages, encompassing a broader range of physicochemical and functional variability than would be achieved using a single species. This strategy provides a more realistic scenario for the potential implementation of portable NIRS as a rapid tool for integrated berry quality assessment. Therefore, the objective of this study was to evaluate the potential of a portable NIR spectrophotometer to develop global PLSR models for the simultaneous prediction of moisture content, total soluble solids, pH, total phenolic content, and antioxidant capacity in aronia, haskap, and goji berries.

## 2. Materials and Methods

### 2.1. Plant Materials

Aronia (*Aronia melanocarpa* (Michx.) Elliott.), haskap (*Lonicera caerulea* L.) and goji berry (*Lycium barbarum* L.) fruits (Figure S1) were harvested during the 2023, 2024, and 2025 growing seasons from plants cultivated at the CICYTEX experimental field in Malpartida de Plasencia (Extremadura, Spain) (39◦54′31.7′′ N, 5◦55′18.4′′ W), which has an inland Mediterranean climate. Fruits from each species were collected at three ripening stages: véraison (onset of ripening), commercial maturity, and overripe. The plant material originated from varietal collections comprising accessions of each species from diverse geographical origins. Plants were cultivated under organic management, employing drip irrigation. Nutrient management consists of a winter compost application supplemented with several foliar treatments of algae-based biostimulants during the growing season. Pest control was carried out using products approved for organic farming, including sulfur, horticultural oils, and natural pyrethrins. Immediately after harvest, the fruits were refrigerated at 2 ± 1 °C and transported to the CICYTEX-INTAEX facilities. Upon arrival, the samples were rapidly frozen and stored at −80 °C in airtight containers to preserve bioactive compounds and minimize moisture loss.

### 2.2. Acquisition of the NIR Spectra

**Figure 1:**
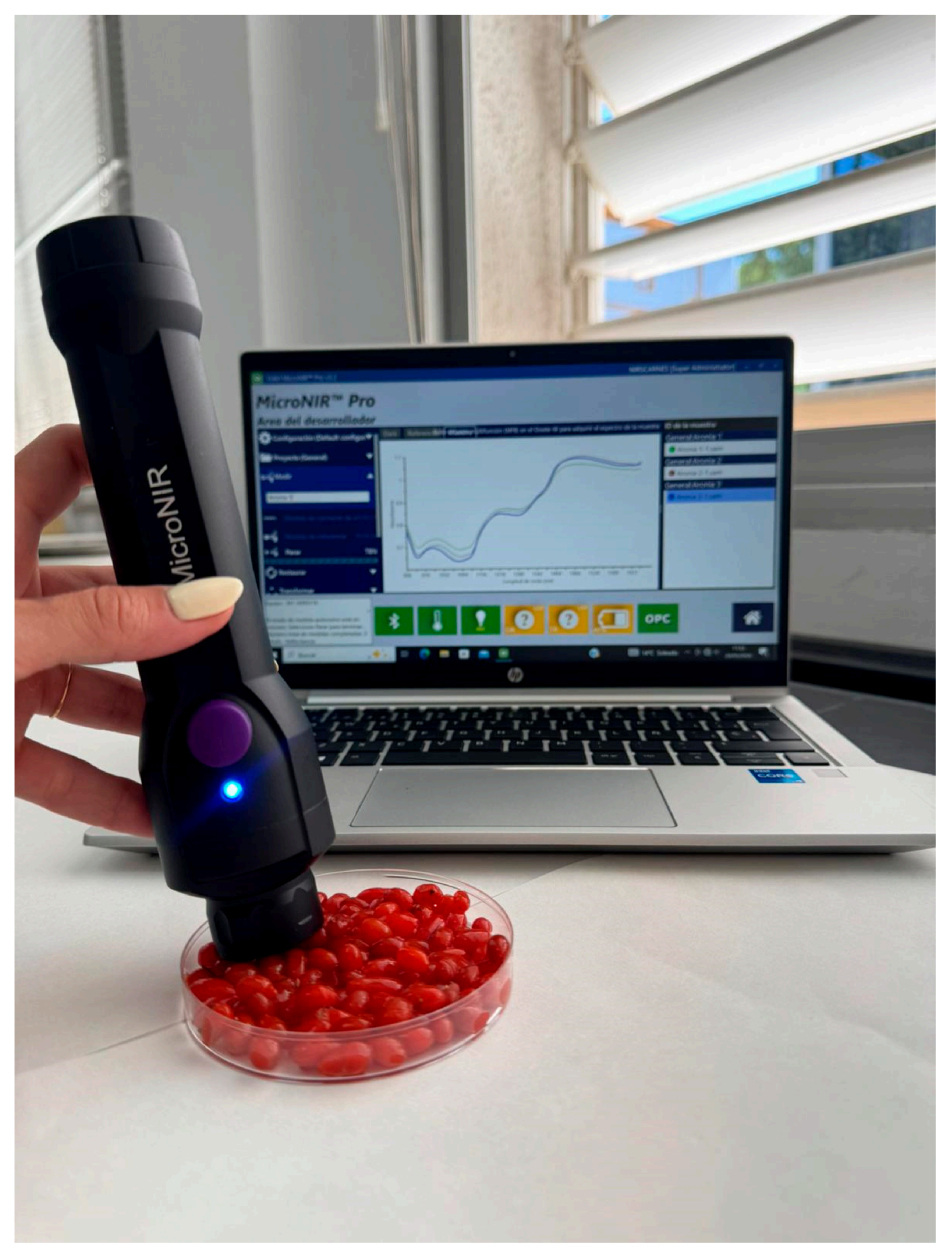
Acquisition of spectral information from berry samples placed in a Petri dish using the portable MicroNIR™ 1700 OnSite-W spectrophotometer (VIAVI Solutions, Inc., San Jose, CA, USA) connected to MicroNIR Pro v2.2 software.

Frozen berry samples (aronia, haskap, and goji) were thawed under refrigerated conditions (4 °C, 1 h) to ensure a uniform temperature throughout the sample while minimizing metabolic and physicochemical changes. The berries were then placed in 90 mm diameter Petri dishes for spectral acquisition. For each measurement, a sufficient number of berries were distributed to completely cover the surface of the Petri dish, ensuring a homogeneous and representative sampling area. Spectra were acquired directly from the thawed berries using a miniaturized portable MicroNIR™ 1700 OnSite-W microspectrophotometer (VIAVI Solutions, Inc., San Jose, CA, USA), configured for in situ analysis (Figure 1). During acquisition, the probe was kept in direct contact with the fruit. Spectral measurements were obtained using a standardized dynamic scanning procedure in which the spectrometer was moved across the berry surface at an approximately constant speed in a zigzag (back-and-forth) pattern. This approach maximized the scanned surface area and captured the natural variability within each sample. The integration time, defined as the detector exposure time for each spectral scan, was set to 10 ms. According to the manufacturer’s specifications, the instrument had a spectral bandwidth of 6.2 nm.

For each independent berry sample placed in a Petri dish, one representative spectrum was obtained using this dynamic sweeping procedure. Thus, the final spectral dataset comprised 145 spectra, corresponding to 145 independent berry samples: 55 aronia, 44 goji, and 46 haskap samples.

Prior to spectral acquisition, the instrument was calibrated following the manufacturer’s recommended procedure. First, a dark reference (“autozero”) was recorded at a fixed location to enable automatic background correction. This procedure continuously adjusts the spectrum’s baseline in real time, minimizing background interferences. Subsequently, a white reference was acquired using a Spectralon™ standard (99% diffuse reflectance) to calibrate the instrument and compensate for signal variations caused by environmental conditions or fluctuations in the light source.

Instrument operation and data acquisition were carried out using MicroNIR Pro v2.2 software. Finally, the spectra, expressed as absorbance [log(1/R), where R represents reflectance], were exported to Unscrambler X v10.5 software—CAMO^®^, Trondheim, Norway—for further processing.

### 2.3. Quantitative NIR Predictive Models

For model development, spectra from aronia, haskap, and goji samples were combined into a single dataset. This combined dataset was used to build global PLSR models for each quality parameter, rather than species-specific models.

Quantitative prediction models were developed for the main physicochemical and functional quality attributes, including total soluble solids (°Brix), moisture content, total phenolic content (mg GAE g^−1^ FW), antioxidant capacity (g Trolox kg^−1^ FW) and pH, using the partial least squares regression (PLSR) algorithm and an internal leave-one-out cross-validation procedure. PLSR reduces the original spectral matrix to a limited number of latent variables (LVs), which are selected to maximize the covariance between the spectral data and the corresponding reference values. The optimal number of LVs was determined as the point beyond which the standard error of cross-validation (SECV) showed no substantial decrease. This approach enables the extraction of relevant chemical information from complex spectral data while minimizing redundant and noise variability [19].

Prior to model development, the complete dataset was divided into two independent groups: a calibration set and an external validation set, in a 70:30 ratio, respectively. The dataset was divided manually and randomly, with the specific aim of ensuring that the three berry species were represented in both sets in a balanced way, as shown in Table 1.

To improve the accuracy of the calibration models, several pre-processing techniques were applied, both individually and in combination. As the objective was to develop global PLSR models, spectra from all berry species were processed together using the same pre-processing procedures, and no species-specific spectral corrections were applied. Standard Normal Variate (SNV) [20] combined with Detrending (DT), and Multiplicative Scatter Correction (MSC) [21] were used to correct scattering effects. In addition, a Savitzky–Golay first-derivative transformation was applied using a symmetric smoothing window with four points on the left and four points on the right side, and a first-order polynomial fit. This treatment is hereafter referred to as SG 1,4,4,1 [22].

The predictive performance of the obtained models was assessed using the coefficient of determination of cross-validation (R^2^CV), the root mean square error of cross-validation (RMSECV), and the number of latent variables (LVs) selected for each model.

Subsequently, the best-fitting predictive models were subjected to external validation. Model performance was assessed by using the coefficient of determination for external validation (R^2^EV) and the root mean square error of external validation (RMSEV). In addition, the residual prediction deviation (RPD) and range error ratio (RER) indices were calculated to evaluate the robustness and practical applicability of predictive models. RPD was calculated as the ratio between the standard deviation of reference values in the external validation set and RMSEV, whereas RER was calculated as the ratio between the range of reference values in the external validation set and RMSEV [23,24].

### 2.4. Physicochemical Analysis

Moisture content, total soluble solids (TSSs), and pH were determined in each independent fruit homogenate corresponding to the samples used for spectral acquisition. Homogenates were prepared using an Omni Mixer homogenizer (Omni International, Marietta, GA, USA). The moisture content was measured gravimetrically by drying approximately 1.0 g of samples at 105 °C for 24 h. TSSs were measured with a Pal-01 digital refractometer (Atago, Tokyo, Japan), and results were expressed as °Brix. pH was determined using a Graphix DL50 titrator (Mettler Toledo, Columbus, OH, USA).

### 2.5. Functional Parameters

Total phenolic content (TPC) was determined according to the method described by Fatchurrahman et al. [16], with slight modifications. Briefly, 1 g of sample was homogenized for 1 min in 30 mL of a water/methanol mixture (20:80, *v*/*v*) containing 2 mmol L^−1^ sodium fluoride using an Omni Mixer homogenizer (Omni International, Marietta, GA, USA). The homogenate was then centrifuged at 9056× *g* for 10 min at 4 °C. For color development, 100 μL of the resulting extract was combined with 1.58 mL of distilled water, 100 μL of Folin–Ciocalteu reagent, and 300 μL of sodium carbonate solution (200 g L^−1^). After incubation for 2 h at room temperature, the absorbance was measured at 725 nm using a UV–Vis spectrophotometer (Shimadzu, Kioto, Japan). TPC was quantified using a calibration curve prepared with gallic acid, and results were expressed as mg gallic acid equivalents per g of fresh weight (mg GAE g^−1^ FW).

Antioxidant capacity was evaluated according to Garbetta et al. [25], with minor modifications. An aliquot of 25 μL of the same extract was mixed with 0.950 mL of DPPH solution. After incubation for 1 h, absorbance was measured at 515 nm. Trolox was used as the reference standard, and results were expressed as g Trolox per kg of fresh weight (g Trolox kg^−1^ FW).

## 3. Results

### 3.1. Composition of Red Berries

The main descriptive statistics for the quality traits measured in red berry samples are presented in Table 2. Overall, the evaluated traits exhibited a wide range of values in both the calibration and validation sets, reflecting the high variability of the sample population. Additionally, similar mean values and standard deviations were observed between the calibration and validation sets for all traits, indicating that the external validation set adequately represented the variability present in the calibration set. The comparable distribution of values in the two sets suggests that the validation samples were sufficiently representative for assessing the performance of the NIRS prediction models.

### 3.2. Spectral Profile

**Figure 2:**
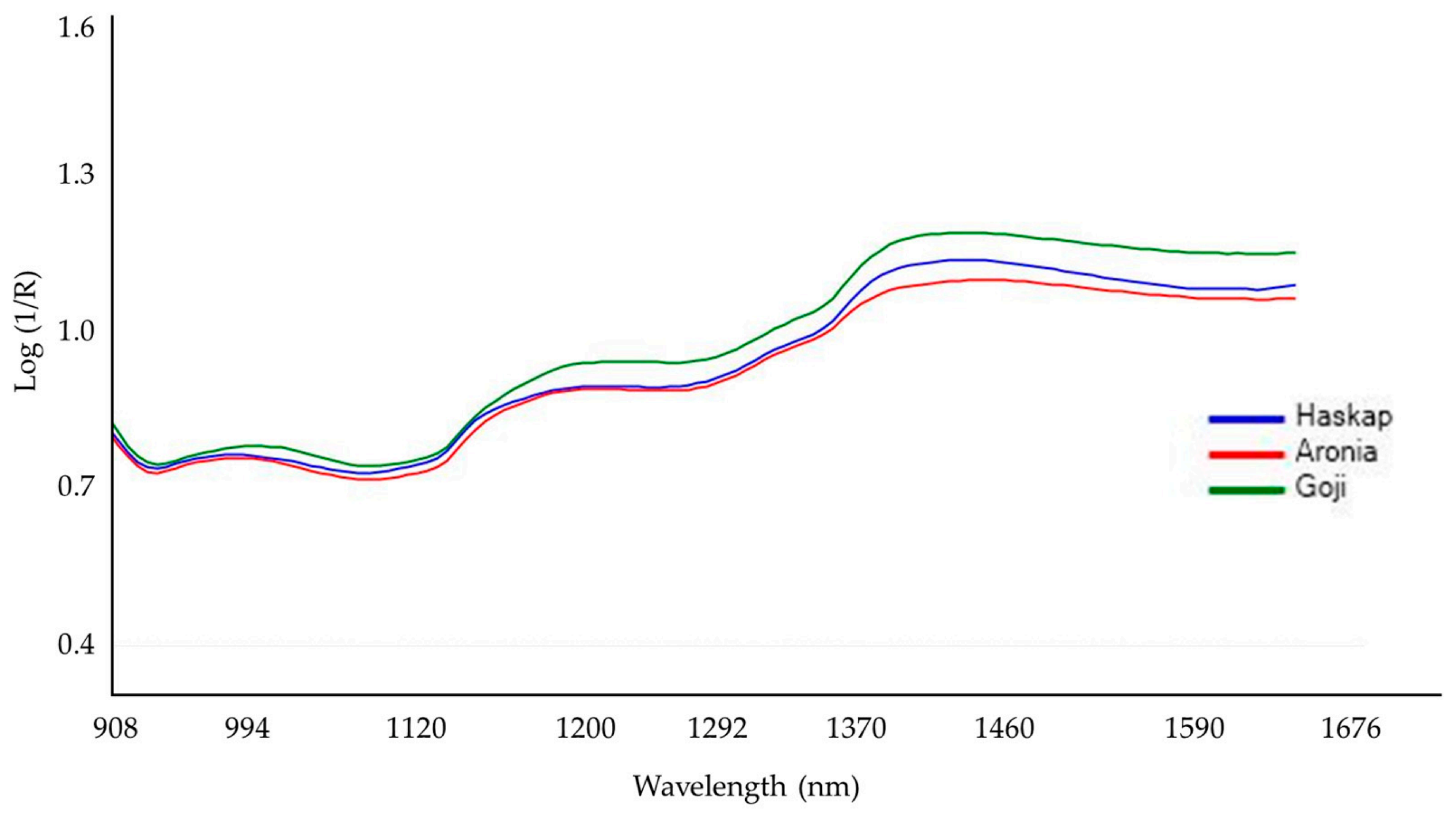
Raw mean spectra expressed as absorbance [log(1/R)] of berry samples grouped according to species: haskap, aronia and goji.

Figure 2 shows the mean raw spectra of the berry samples grouped by species. In general, haskap, aronia, and goji berries exhibited a similar spectral pattern across the entire wavelength range, although differences in absorbance intensity were observed among species. Overall, the spectra showed the typical profile of fruit matrices with high water content, with the most relevant spectral features located around 930–950 nm, 1200–1290 nm, and 1370–1460 nm. The region around 930–950 nm can be mainly associated with the second overtone of O–H stretching, while the shoulder observed between 1200 and 1290 nm may be related to water-associated O–H absorptions and overlapping C–H absorptions from carbohydrates and other organic compounds. The most intense broad absorption band, located approximately between 1370 and 1460 nm, corresponds mainly to the first overtone of O–H stretching, confirming the strong contribution of water to the spectral response of the berry samples [26]. This latter region showed the clearest differences in absorbance among species, with goji berries showing the highest absorbance values, aronia berries showing the lowest, and haskap berries exhibiting intermediate values. These differences may reflect variations among species in water content, soluble solids, and other hydroxyl-containing constituents. Beyond the region of maximum absorbance, the spectra decreased slightly and tended to stabilize toward the end of the spectral range, between 1590 and 1676 nm.

### 3.3. NIR Quantitative Predictive Models

The results of the best-fitting PLSR models developed for the prediction of the main quality traits in red berry samples are shown in Table 3. In addition, the PLSR results obtained using the different spectral pretreatments are provided in the Supplementary Material (Table S1).

In the calibration step, R^2^CV values ranged from 0.83 to 0.92. The highest value was obtained for °BRIX, followed by pH and moisture content, while total phenolic content and antioxidant capacity showed similar R^2^CV values. RMSECV values ranged from 0.281 for pH to 2.085 for antioxidant capacity. After external validation, the predictive ability of the models remained relatively stable, with R^2^EV values ranging from 0.76 to 0.88. The highest value was observed for °BRIX, while moisture content, antioxidant capacity and pH also reached R^2^EV values above 0.80. Total phenolic content showed the lowest validation statistics among the evaluated traits.

Regarding spectral pre-treatments, most of the selected equations were obtained after correcting scattering effects, mainly by applying SNV combined with de-trending, either alone or together with the first derivative Savitzky–Golay treatment. More specifically, the models for °BRIX and pH were developed using SNV + DE + SG 1.4.4.1, whereas the models for total phenolic content and antioxidant capacity were selected after SNV + DE. In contrast, the best model for moisture content was obtained using MSC combined with SG 1.4.4.1.

## 4. Discussion

The descriptive statistics of the samples used for model development and validation adequately reflected the variability considered in this study. This variability was deliberately enhanced by including fruits from different species, harvest years, and stages of ripeness. This approach differs from most previous studies on berries, which have generally focused on individual species, such as goji [16], strawberry [27] or blueberry [18]. By incorporating a broader source of variability, the present study provides a more realistic context for developing global predictive models intended for the integrated assessment of red berry quality.

Regarding spectral information, the interpretation of the NIR spectra should be considered in relation to the dominant role of water in berries, since water is the major constituent of these matrices and the strongest absorber within the spectral range evaluated [26,28]. The spectral region showing the highest absorbance values was located approximately between 1370 and 1460 nm. These bands have been associated with the first overtone of O–H stretching and, therefore, with water absorption, although minor overlapping contributions from other hydroxyl-containing compounds may also be present in this absorption region [16,27]. The second most relevant peak in terms of absorbance intensity, located around 1200 nm, may also be interpreted as water-related, although minor overlapping contributions from C–H bonds associated with carbohydrates and other organic compounds may be present [16,27]. Moreover, the spectral differences observed among the three species, particularly in these regions, may reflect variations in the aforementioned components. The inclusion of this variability in the calibration dataset may have contributed to improving the robustness and applicability of the developed models.

As for the quantitative models, the good predictive performance obtained for °Brix, moisture content, and pH was to some extent expected, since these parameters are closely related to the main components of these fruits. Specifically, °Brix and moisture content are directly associated with soluble solids and water content, respectively, and both significantly influence the spectral response through the O–H and C–H vibrational bands. In the case of pH, although it is not a direct chemical component, its prediction can be explained indirectly by its relationship to the balance between soluble compounds and organic acids.

The predictive ability of the models may also be partly explained by indirect spectral information related to water–solute interactions. In high-moisture matrices such as berries, sugars, organic acids, and phenolic compounds can modify the hydrogen-bonding structure of water, thereby affecting water-dominated NIR regions, particularly around 1430–1460 nm. Thus, these spectral changes may contribute to the prediction of soluble solids and other quality-related traits, even when these compounds are not directly detected through isolated absorption bands [29,30].

The performance of these models was consistent with that reported by Fatchurrahman et al. [16], who used hyperspectral imaging in the Vis–NIR region, specifically between 400 and 1000 nm, to predict quality traits in fresh goji berries (*Lycium barbarum* L., cv. Sweet Berry). These authors obtained external-validation coefficients of determination of up to 0.94 for soluble solids content and 0.84 for titratable acidity. Similar results have also been reported for other berry fruits. Specifically, Seki et al. [17] evaluated the use of Vis–NIR spectroscopy to predict soluble solids content in white and red strawberries, using spectral ranges of 500–978 nm and 908–1676 nm, respectively, obtaining coefficients of determination above 0.85. Likewise, Qiu et al. [18] used NIR hyperspectral imaging in the 900–1700 nm range to determine soluble solids content in fresh blueberries, obtaining coefficients of determination for prediction above 0.86. In the present study, comparable predictive accuracy was obtained despite the greater heterogeneity of the sample set, which included three berry species, samples from different harvest years, and several ripening stages.

On the other hand, the comparatively lower predictive performance observed for the functional parameters, particularly total phenolic content, may be attributed to the lower concentrations of the compounds involved and the fact that their spectral contribution is often masked by the more intense absorption bands of major constituents, such as water and soluble carbohydrates. Consequently, their prediction relies more strongly on the ability of the chemometric models to extract subtle spectral variations associated with these compounds from the overall spectral response. Moreover, both total phenolic content and antioxidant capacity are complex parameters, as they are not associated with a single chemical compound but with the combined contribution of several bioactive molecules, whose spectral response may vary depending on the species, maturity stage and individual composition of the fruit matrix. In any case, all models maintained high coefficients of determination after external validation. This finding is consistent with previous studies on berries, in which the prediction of bioactive-related parameters has generally been more challenging than the prediction of major constituents, such as soluble solids or acidity-related traits. For instance, Fatchurrahman et al. [16] reported limited external predictive performance for total phenolic content in fresh goji berries, with an R^2^pred of 0.62, while the prediction of total antioxidant activity and anthocyanin content yielded unsatisfactory results. Mancini et al. [27], working with strawberry cultivars, highlighted the usefulness of NIR spectroscopy for the rapid assessment of nutritional quality, but approached bioactive compounds such as anthocyanins and phenolic acids mainly through cultivar discrimination rather than direct quantitative prediction. In contrast, higher predictive ability has been reported for dried black goji berries (*Lycium ruthenicum* Murr.), for which Zhang et al. [31] applied NIR hyperspectral imaging to determine total phenolics, flavonoids and anthocyanins, obtaining an R^2^pred of 0.84 for total phenolics. This higher performance may be partly explained by the dried nature of the samples, which increases the relative concentration of phenolic compounds and reduces the dominance of water absorption in the spectra, potentially facilitating the extraction of spectral information related to these bioactive compounds. More recently, Hu et al. [32] combined hyperspectral imaging with an advanced deep learning model to simultaneously predict polysaccharides, polyphenols and carotenoids in goji berries, achieving higher coefficients of determination than those observed in the present study for these bioactive components. However, this approach relies on hyperspectral imaging and complex computational modelling, whereas the present study used a miniaturized portable NIR spectrophotometer, which is simpler, faster and more suitable for potential on-site or industrial applications.

The limited decrease in predictive performance observed from cross-validation to external validation further supports the stability of the developed models. This reduction ranged from only 1.20% for antioxidant capacity to 8.43% for total phenolic content, with intermediate decreases of 4.35% for °Brix, 5.62% for moisture content, and 7.78% for pH. Such a moderate loss of predictive performance suggests that the models were not substantially overfitted and that the variability of the sample population was adequately represented in both the calibration and external validation sets. In addition, the RPD and RER statistics reinforce the practical relevance of the models. According to commonly used interpretation criteria for NIR calibration models, RPD values between 2.0 and 2.5 are generally considered suitable for screening or approximate quantitative prediction, whereas values above 2.5 indicate moderate-to-good predictive performance. Similarly, RER values between 6 and 10 are usually considered suitable for approximate prediction, whereas values between 10 and 15 indicate fair quantitative predictive ability. In the present study, RPD values ranged from 2.02 to 2.89, while RER values ranged from 6.82 to 9.70, indicating that the developed models were mainly suitable for approximate quantitative prediction [24,33].

## 5. Conclusions

The results of the present study demonstrate the potential of portable NIR spectroscopy for the rapid and non-destructive assessment of physicochemical and functional quality traits in emerging red berries species. The global PLSR models developed using spectral data acquired from aronia, haskap and goji berries using a miniaturized NIR spectrophotometer showed predictive performance suitable for the approximate quantitative prediction of °Brix, moisture content, pH, total phenolic content and antioxidant capacity. These prediction models could represent an initial step towards the implementation of portable NIRS technology as a practical tool for the integrated quality assessment of these emerging crops. The inclusion of fruits from different species, harvest years and ripening stages allowed the calibration models to capture a broad range of natural variability, which may enhance their robustness and applicability under diverse production conditions. Combined with the use of a portable instrument, this approach could support fruit ripening monitoring in the field and quality assessment throughout the value chain. Although the models showed satisfactory predictive performance for frozen–thawed berry samples, their direct transferability to fresh, intact fruits requires further evaluation under field or industrial conditions.

## Figures and Tables

**Table 1:** Distribution of samples in the calibration and validation sets according to species.

Species	Calibration	Validation	Total
Aronia	41	14	55
Goji	31	13	44
Haskap	29	17	46
Total	101	44	145

**Table 2:** Descriptive statistics of quality traits of red berry samples.

Parameter	Calibration				Validation			
	Mean	Min	Max	SD	Mean	Min	Max	SD
°Brix	16.07	8.80	24.40	4.03	16.04	9.20	22.60	4.03
Moisture (%)	82.38	72.28	93.51	5.16	82.67	73.05	91.40	5.34
mg GAE g^−1^	7.50	1.81	17.61	4.48	7.25	1.84	17.61	4.59
g Trolox kg ^−1^	6.65	0.54	17.95	5.16	6.19	0.54	17.95	4.86
pH	4.06	2.73	5.59	0.91	4.08	2.73	5.55	0.93

Min = minimum observed value; Max = maximum observed value; SD = standard deviation.

**Table 3:** PLSR results of the best fitting prediction models for the main quality traits of red berry samples.

Parameter	Pre-Treatment	Calibration				External Validation				
		*n*	LVs	R^2^CV	RMSECV	*n*	R^2^EV	RMSEV	RPD	RER
°Brix	SNV + DE + SG 1.4.4.1	93	12	0.92	1.129	44	0.88	1.391	2.89	9.70
Moisture (%)	MSC + + SG 1.4.4.1	100	11	0.89	1.450	42	0.84	2.095	2.49	8.75
mg GAE g^−1^ FW	SNV + DE	95	5	0.83	1.757	43	0.76	1.914	2.02	6.82
g Trolox kg^−1^ FW	SNV + DE	93	12	0.83	2.085	40	0.82	1.827	2.26	9.53
pH	SNV + DE + SG 1.4.4.1	96	6	0.90	0.281	44	0.83	0.381	2.44	7.40

SNV = standard normal variate; DE = de-trending; MSC = multiplicative scatter correction; SG = Savitzky–Golay derivatives, where the first number indicates the derivative order, the second and third numbers indicate the number of smoothing points on the left and right sides, respectively, and the last number corresponds to the polynomial order; LVs = latent variables; R^2^CV= coefficient of determination in cross-validation; RMSECV = root mean square error of cross-validation; R^2^EV = coefficient of determination in external validation; RMSEV = root mean square error of validation; RPD = residual prediction deviation in validation; RER = range error ratio in validation. RMSECV and RMSEV are expressed in the same units as each corresponding reference parameter: °Brix for total soluble solids, % for moisture, mg GAE g^−1^ FW for total phenolic content, g Trolox kg^−1^ FW for antioxidant activity, and pH units for pH.

## Data Availability

The original contributions presented in the study are included in the article/Supplementary Materials; further inquiries can be directed to the corresponding author.

## Supplementary Materials

The following supporting information can be downloaded at: https://www.mdpi.com/article/10.3390/foods15152679/s1, Figure S1: Representative fruits of goji (*Lycium barbarum*), haskap (*Lonicera caerulea*), and aronia (*Aronia melanocarpa*). Table S1: PLSR results of the prediction models for the main quality traits of berry samples according to spectral pre-treatment.
