# Supplementary material for: NIR Spectroscopy for Predicting Physicochemical and Functional Quality Attributes of Berry (Aronia, Haskap, and Goji) Fruits
Source: Foods. 2026 Jul 29;15(15):2679. doi: 10.3390/foods15152679 (PMC13464982; doi:10.3390/foods15152679)
Supplement: Supplementary file 1 [file foods-15-02679-s001.zip › Table S1.pdf]

Table S1. PLSR results of the prediction models for the main quality traits of berry samples according to spectral pre-treatment

Parameter	Pre-treatment	Cross-validation			
		**n**	**LV**	**1-VR**	**RMSECV**
BRIX	Abs	91	12	0,87	1.482
	MSC	96	12	0,89	1.360
	SNV + DE	99	12	0,89	1.263
	SNV + DE + SG 1,4,4,1	93	12	0,92	1.129
Moisture (%)	Abs	93	12	0,87	1.816
	MSC	94	11	0,88	1.747
	MSC + + SG 1,4,4,1	100	11	0,89	1.450
	SNV + DE	92	7	0,85	1.921
mg GAE g^{-1} FW	Abs	91	8	0,81	1.913
	MSC	87	9	0,79	1.969
	SNV + DE	95	5	0,83	1.757
	SNV + DE + SG 1,4,4,1	91	4	0,84	1.715
mg TE kg^{-1} FW	Abs	89	8	0,70	2.766
	MSC	94	12	0,79	2.444
	SNV + DE	93	12	0,83	2.085
	SNV + DE + SG 1,4,4,1	99	9	0,76	2.536
pH	Abs	95	10	0,88	0,301
	MSC	95	8	0,85	0,349
	SNV + DE	100	8	0,89	0,303
	SNV + DE + SG 1,4,4,1	96	6	0,90	0,281
