# Supplementary material for: NIR Spectroscopy for Predicting Physicochemical and Functional Quality Attributes of Berry (Aronia, Haskap, and Goji) Fruits
Source: Foods. 2026 Jul 29;15(15):2679. doi: 10.3390/foods15152679 (PMC13464982; doi:10.3390/foods15152679)
Supplement: Supplementary file 1 [file foods-15-02679-s001.zip › Figure S1 Supplementary material.pdf]

a) b) c)

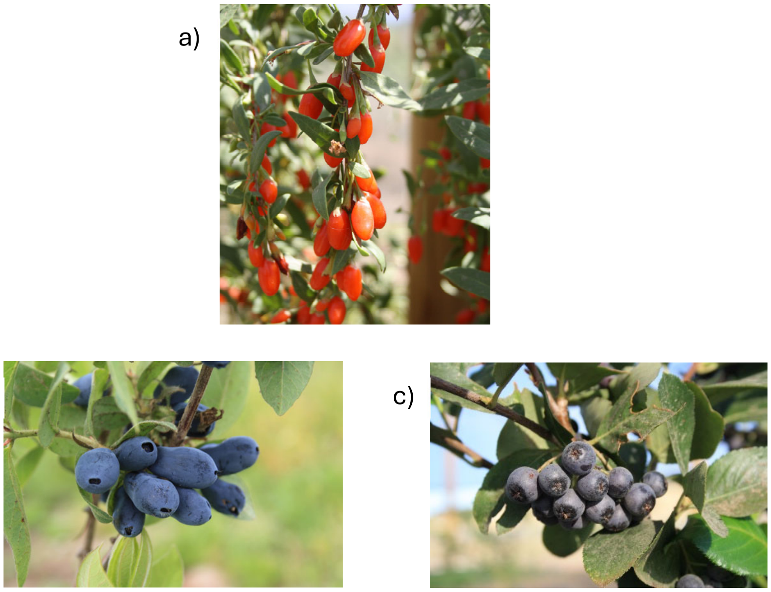

Figure S1. Representative fruits of goji (*Lycium barbarum*) (a), haskap (*Lonicera caerulea*) (b), and aronia (*Aronia melanocarpa*) (c).
